# HIV testing in Europe: Evaluating the impact, added value, relevance and usability of the European Centre for Disease Prevention and Control (ECDC)’s 2010 HIV testing guidance

**DOI:** 10.2807/1560-7917.ES.2017.22.48.17-00323

**Published:** 2017-11-30

**Authors:** Ann K Sullivan, Ida Sperle, Dorthe Raben, Andrew J Amato-Gauci, Jens Dilling Lundgren, Yazdan Yazdanpanah, Stine Finne Jakobsen, Lara Tavoschi

**Affiliations:** 1 Chelsea and Westminster Hospital NHS Foundation Trust, London, United Kingdom; 2 CHIP, Rigshospitalet, University of Copenhagen, Denmark; 3 European Centre for Disease Prevention and Control (ECDC), Stockholm, Sweden; 4 INSERM, Paris, France

**Keywords:** HIV, Testing, Guidance, evaluation, EU/EEA

## Abstract

Background: An evaluation of the 2010 ECDC guidance on HIV testing, conducted in October 2015–January 2016, assessed its impact, added value, relevance and usability and the need for updated guidance. Methods: Data sources were two surveys: one for the primary target audience (health policymakers and decision makers, national programme managers and ECDC official contact points in the European Union/European Economic Area (EU/EEA) countries and one for a broader target audience (clinicians, civil society organisations and international public health agencies); two moderated focus group discussions  (17 participants each); webpage access data; a literature citation review; and an expert consultation (18 participants) to discuss the evaluation findings. Results: Twenty-three of 28 primary target audience and 31 of 51 broader target audience respondents indicated the guidance was the most relevant when compared with other international guidance. Primary target audience respondents in 11 of 23 countries reported that they had used the guidance in development, monitoring and/or evaluation of their national HIV testing policy, guidelines, programme and/or strategy, and 29 of 51 of the broader target audience respondents reported having used the guidance in their work. Both the primary and broader target audience considered it important or very important to have an EU/EEA-level HIV testing guidance (23/28 and 46/51, respectively). Conclusion: The guidance has been widely used to develop policies, guidelines, programmes and strategies in the EU/EEA and should be regularly updated due to continuous developments in the field in order to continue to serve as an important reference guidance in the region.

## Introduction

According to the 2015 joint European Centre for Disease Prevention and Control (ECDC) and World Health Organization (WHO) HIV surveillance report, rates of HIV testing among populations most at risk of HIV in the WHO European Region were low overall, and hence, a considerable proportion of people who are infected with HIV are not aware of being infected [1]. This means that many who need treatment are not receiving it because they have not been diagnosed and linked to care. In addition, rates of late diagnosis of HIV were high at 47%, among newly diagnosed in 2015 which has not changed significantly in the European Union/European Economic Area (EU/EEA) since 2010 [1,2]. Earlier diagnosis enables people to start treatment early, which increases their chances of normal life expectancy and healthier life while reducing the risk of onward transmission [1].

Increasing the availability, accessibility and uptake of HIV testing is critical to reduce the number of people who do not know their HIV status or who are diagnosed late. With the ambitious goals of 90–90–90 set by the UNAIDS, the first 90 means that 90% of people living with HIV should know their status [3]. However, targeting HIV testing programmes to those who are most at risk remains a challenge in many countries in the EU/EEA. Monitoring HIV testing coverage is patchy and heterogeneous, making it hard to assess trends over time at EU/EEA level and to provide useful data to inform action [4]. A small increase in the total number of tests performed has been seen overall, while some countries have shown larger increases in testing [5].

ECDC published a commissioned guidance in 2010 [6], together with the literature review that provided the evidence base [7]. The guidance intended to inform the development, monitoring and evaluation of national HIV testing strategies and programmes in the EU/EEA. It included the following topics: (i) core principles for national HIV testing strategies and programmes, (ii) developing a national HIV testing strategy, (iii) ensuring access to HIV treatment, care and prevention, and (iv) monitoring and evaluation. Its target audience was health policymakers and decision makers in EU/EEA countries, national programme managers and coordinators, ECDC national focal points (nominated ECDC contact points responsible for strategic and operational collaboration on technical and scientific issues) and disease network experts. Since 2010, the HIV testing landscape has evolved with new evidence and results for example on indicator condition guided HIV testing [8,9], when to start treatment, self-testing and home sampling, and additional guidelines have subsequently been published, some more up to date such as those by WHO in 2015 [10], the International Union against Sexually Transmitted Infections (IUSTI) [11] and the European Monitoring Centre for Drugs and Drug Addiction (EMCDDA) [12].

Our aim was to evaluate the use and impact of the ECDC HIV testing guidance in the EU/EEA in order to make recommendations for future steps by ECDC in this area, including the potential need for an updated guidance. Our objective was to assess (i) awareness of the guidance among the primary target audience and the broader target audience, (ii) the relevance and usability of the guidance for the primary target audience and the broader target audience, (iii) the extent to which the guidance has added value to or complemented existing documents and (iv) the impact of the guidance on supporting the development, monitoring and evaluation of national HIV testing strategies or programmes in the countries.

## Methods

The evaluation was conducted between October 2015 and January 2016. Data were collected through: two evaluation surveys; two moderated focus group discussions; webpage access data; a literature citation review; and an expert consultation hosted by ECDC to discuss the evaluation findings as well as to validate and interpret the results. The two surveys were one for the primary target audience for the guidance, consisting of health policymakers and decision makers, national programme managers and ECDC official contact points in the 31 EU/EEA countries and one for the broader audience, including clinicians, civil society organisations and international agencies.

### Evaluation surveys

Survey questions were designed to address the aims and objectives of the evaluation. To contextualise the use and impact of the guidance, the surveys asked about existing national HIV testing guidelines, programmes and services. They included questions with both pre-defined answer categories and free text fields to allow the respondents to qualify and further explain their answers. The two surveys were similar except for five questions on national-level HIV testing policies and programmes which were not included in the one to the broader audience. Surveys were set up in a secure, web-based application designed to support data capture for research studies (REDCap). They were piloted across five countries. Five people piloted the survey for the primary target group (from Estonia, Greece, Norway and the United Kingdom (UK)) and three the survey for the broader target group (from Spain and UK). The feedback was incorporated into the final versions of the two surveys.

Primary target audience respondents were identified using a purposive sampling approach to ensure that there was at least one respondent from each EU/EEA country. Potential respondents were identified by the official ECDC Coordinating Competent Bodies and National Focal Points for HIV in each country, with priority given to policymakers, advisors and technical experts at a national level with responsibility for and expertise in HIV testing and guidance development and implementation. For the countries that did not nominate respondents, the survey was sent directly to National Focal Points for HIV. The broader audience survey was distributed widely through mailing lists of HIV organisations and professional networks and sent to individual experts known to be working in the field of HIV. The target was to receive 150 responses.

The survey data were extracted in Excel format from REDCap and descriptive statistics were produced as frequencies and respective proportions in Excel.

### Focus group discussions

Two focus group discussions of one hour each were conducted in October 2015 during the European AIDS Clinical Society (EACS) conference in Barcelona, Spain. A total of 17 participants from six countries took part in the two discussions. A semi-structured interview guide was developed to lead the focus group discussions and ensure coverage of the evaluation objectives (e.g. Do you consider there is added value at EU/EEA level of having an ECDC HIV testing guidance? If so, in what way?). The data were analysed using a deductive approach and the research questions were the basis for grouping data according to themes, similarities and differences, resulting in a descriptive content analysis.

### Webpage access data and review of literature citation

An analysis of traffic related to the page on the ECDC website hosting the guidance was performed in January 2016. The analysis covered the period from January 2014 and onwards due to a platform migration of ECDC landing page in 2013. It captured the number of page views, sources of traffic and access country.

Citation screening was conducted to identify relevant citations of the guidance. Searches in Scopus and Google Scholar were performed on 8 January 2016 to retrieve articles and documents in all languages citing the guidance in the period December 2010 to end December 2015 using words of the title in the ‘references’ field and parts of the URL in the ‘website’ tag in the advanced search in Scopus.

### Expert consultation

An Expert Panel was established by ECDC to provide expert opinion on the validation, interpretation and presentation of the findings from the evaluation and to contribute to the identification of priorities for actions and next steps for ECDC in the area of HIV testing in the EU/EEA. The Expert Panel consisted of 18 members from 14 countries, representing various constituencies including national public health bodies, clinical societies, academia, civil society and international organisations.

## Results

### Evaluation surveys

Twenty-eight respondents from 23 of 31 EU/EEA MS completed the primary target audience survey (Figure). Five countries submitted surveys from two respondents. Where the answers were conflicting, responses were analysed in more depth, taking into account the background of the respondents. Fifty-one respondents from 18 EU/EEA countries and one multinational organisation (WHO) completed the survey for the broader audience (Figure).

**Figure:**
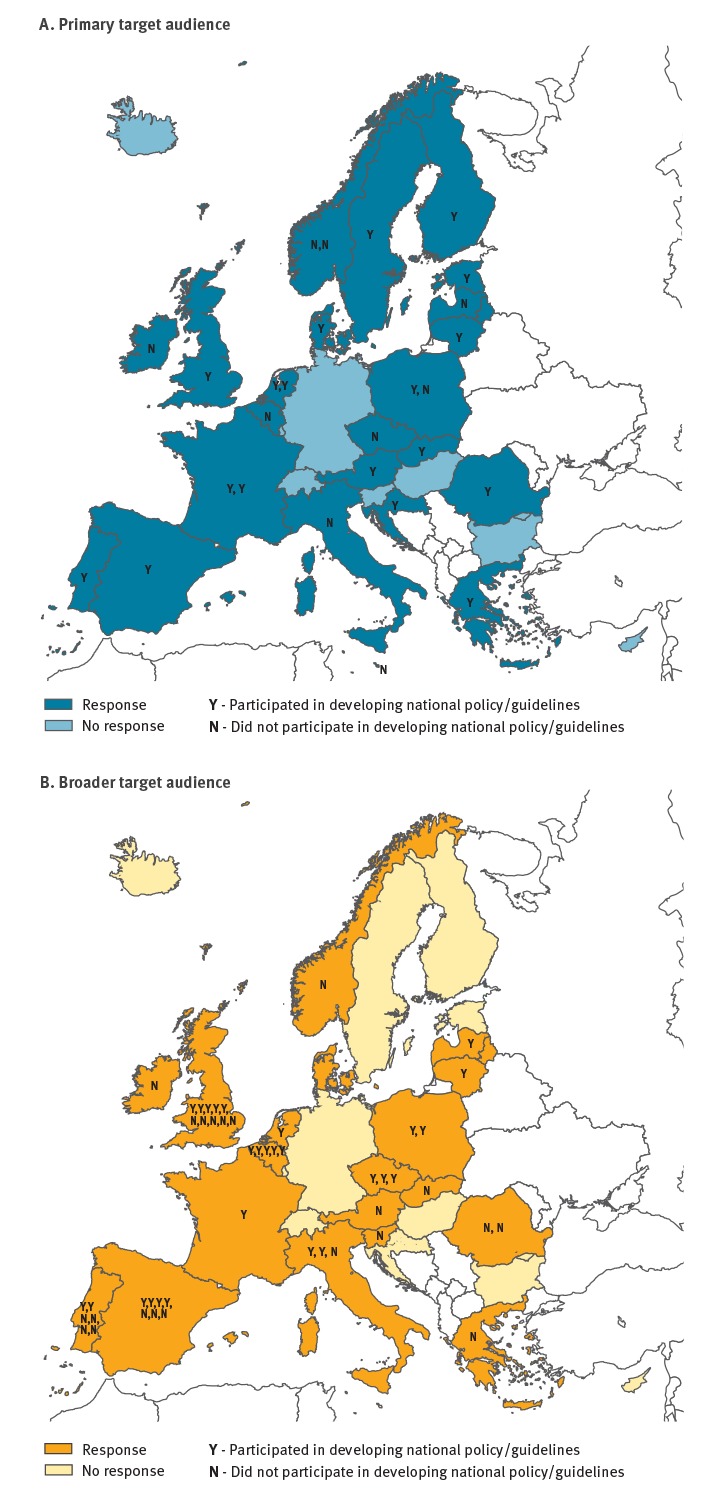
Country location of survey respondents and their role in developing national HIV policy/guidelines, ECDC HIV testing guidance evaluation, October 2015–January 2016 (n=23 EU/EEA countries)

ECDC: European Centre for Disease Prevention and Control; EU/EEA: European Union/European Economic Area.

Total of N/Y indicates the number of individual responses in panel A and number of responses by multinational organisations in panel B.

#### National guidelines and programmes

The primary target audience provided background information on the general use of national reference documents on testing. The most used documents were national HIV strategy/policy documents that include recommendations on testing (18/28), national HIV testing guidelines (14/28) and HIV testing guidelines issued by professional societies (13/28). Different levels of HIV testing programmes were reported by the respondents (Table 1).

**Table 1:** HIV testing in the countries of the primary target audience respondents, ECDC HIV survey, October 2015–January 2016 (n = 23 EU/EEA countries)

Characteristics of HIV testing policy and practices	Total (n=23)
National HIV testing programme	7
Including national HIV testing monitoring and evaluation plan	4^a^
Testing programmes and services at sub-national level	7^a^
No national HIV testing programme but health system that conducts HIV testing	9
**Elements included in HIV testing practices based on reported frequency (most and least)**	
Post-test access to treatment, care and prevention services	17
Voluntary, confidential testing with informed consent	16
Testing of all pregnant women for HIV (opt-out)	16
Dedicated HIV testing centres (e.g. for people at high risk, PWID services)	15
Routine offering in emergency departments	4
Written informed consent	2
Home testing/self-testing	1

ECDC: European Centre for Disease Prevention and Control; EU/EEA: European Union/European Economic Area; PWID: people who inject drugs.

^a^ Only those seven reporting having a national HIV testing programme were asked this question.

### Awareness of the ECDC HIV testing guidance

All 28 primary target audience respondents and 42 of 51 respondents of the broader target audience respondents were aware of the ECDC HIV testing guidance.

The guidance had a total of 619 page views (530 unique views) on the ECDC website and was cited in the literature 79 times: 74 times in scientific journals (original articles (n=65), reviews (n=4), editorials (n=3), commentaries (n=1) letters (n=1)), three times in reports, and once in a book and in a thesis, respectively. In six of these, ECDC staff was a first author. Half of the citations (40/79; 51%) reported or referenced the recommendations or core principles in the guidance.

### Relevance and usability of the guidance

Twenty-three of the 28 primary target audience respondents and 31 of the 51 broader target audience respondents indicated the guidance as being the most relevant in their respective context when compared with other international guidance such as the WHO 2015 guidelines [10], IUSTI 2014 guidelines [11] and EMCDDA 2010 guidance [12]. The majority reported that the guidance was relevant when it was published in 2010 and that it remains relevant today.

For the primary target audience, the guidance was mainly considered relevant as a reference policy document (18/21), for use in national HIV testing policy/guidelines/strategy development, monitoring and/or evaluation (16/21), and for general information on approaches to HIV testing (16/21).

For the broader target audience, the guidance was mainly considered relevant as a reference policy document (19/30), for general information on approaches to HIV testing (17/30), and for support for advocacy work on HIV testing to influence decision makers and raise awareness (16/30).

In terms of usability, the majority of both groups of respondents strongly agreed or agreed that the guidance is (i) user-friendly (primary target audience: 16/23; broader target audience: 33/51), (ii) clearly written and easy to understand (primary target audience 22/23; broader target audience 39/51), (iii) easily accessible (primary target audience 21/23; broader target audience 31/51) and (iv) includes enough details about HIV testing (primary target audience 16/23; broader target audience 31/51).

### Added value of the guidance

Twenty-three of 28 primary target audience respondents and 46 of 51 broader target audience respondents considered it very important or important to have EU level HIV testing guidance (Table 2).

**Table 2:** Importance of having an EU/EEA-level guidance by target audience, ECDC HIV survey, October 2015–January 2016 (n = 23 EU/EEA countries)

Importance of having an EU/EEA- level guidance	Primary target audience (n=28)	Broader target audience (N=51)
Very important	9	28
Important	14	18
Somewhat important	2	4
Not important	1	1
No response	1	0

ECDC: European Centre for Disease Prevention and Control; EU/EEA: European Union/European Economic Area.

Respondents highlighted a number of ways in which they saw the ECDC guidance to add value and to influence change in individual countries. For the primary target audience, the guidance mainly adds value by providing a well-recognised policy reference document (18/25), by providing an EU standard (15/25) and by saving time and resources as it provides an up to date review of evidence that is relevant to the EU/EEA (15/25).

For the broader target audience, the main added value of the guidance is influencing the development of national policies (35/50).

### Use and impact of the guidance

Almost half of the countries (10/23) reported having used the guidance in the development, monitoring and/or evaluation of their national HIV testing policy/guidelines/programme/strategy. Of the broader target audience respondents, 29 of 51 reported having used the guidance for their work. The respondents who reported having used the guidance were then asked to report how they had used it (Table 3).

**Table 3:** Reported use of the ECDC HIV testing guidance of those who reported guidance use by target audience, ECDC HIV testing guidance evaluation, October 2015–January 2016 (n = 23 EU/EEA countries)

Use of the guidance	Primary target audience (n = 10)	Broader target audience (n = 29)
To revise an existing HIV testing document	6	19
To support/inform the monitoring/evaluation of HIV testing	5	9
To develop a new HIV testing document	3	12
To advocate for HIV testing, raise awareness of HIV testing	3	18
To fundraise/mobilise resources for HIV testing programmes	2	13
Other	2	1
To influence decision makers	NA	4

ECDC: European Centre for Disease Prevention and Control; EU/EEA: European Union/European Economic Area; NA: not applicable.

Twenty of the 28 primary target audience respondents and 38 of the 51 broader target audience respondents reported changes in HIV testing practices in their country since 2010. Of these, 16 of 20 and 16 of 38, respectively, reported that in their opinion these changes led to an improvement in HIV testing in their country. The guidance was considered as having directly influenced these changes by seven of the 20 primary target audience respondents and nine of the 38 broader target audience respondents.

### Updating and revising the guidance

Twenty-six of 28 respondents highlighted at least one new area/topic that should be added in a new guidance document. The need to update the HIV testing guidance due to significant new developments within the HIV testing field, was also confirmed in the focus group discussions. An updated guidance should have a greater focus on monitoring and evaluation of HIV testing programmes and services and include examples of best practice to help foster effective implementation. In addition, focus group discussions highlighted the fact that future testing guidance should also embrace a broader target audience, to also take account of those involved in guidance development and implementation within and outside of the EU/EEA.

## Discussion

This study attempted to assess the impact of the ECDC HIV testing guidance 5 years after its release, using a multidisciplinary approach. The findings indicate there is a high level of awareness of the guidance, that it has reached a wider audience than the intended audience, that it is perceived to be relevant and useful, and that it adds value by providing an EU/EEA-wide perspective. The guidance has been widely used to develop and revise national policies, guidelines, programmes and strategies as well as to support monitoring and evaluation and advocacy, and has contributed to changes in HIV testing strategies in a number of EU/EEA countries.

There is, however, a lack of data on HIV testing coverage and uptake, and of standardised data in particular. This makes it difficult to accurately measure changes in testing across the EU/EEA over time but the limited improvements described in testing rates and in the number of late presenters [13] suggest that testing policies and guidelines are not being implemented effectively. For example, the ECDC 2010 guidance emphasises the need for scaling up indicator condition guided HIV testing and community-based testing, which are the focus of a number of recent initiatives and projects in Europe, including the EU funded OptTEST [14], Euro HIV EDAT and COBATEST [15] projects.

Despite this, recent data show that indicator condition guided HIV testing [8,16] is not being widely implemented in Europe. The OptTEST project has reviewed national HIV testing guidelines and audited implementation of indicator condition guided HIV testing in seven pilot countries. The results show low rates of HIV testing in patients presenting with indicator conditions as well as sub-optimal inclusion of indicator condition guided testing recommendations both in national HIV testing guidelines and relevant specialty guidelines [17,18].

Community-based testing has been expanded in the EU/EEA since 2010 through a variety of service delivery models [19]. However, limited funding, poor integration with national HIV programmes and regulatory barriers in some cases (for example, restricting testing to trained health professionals), have hindered further scale up [4,20]. These findings reinforce the need for guidance to clearly address these implementation challenges, including through direct guidance in relation to engagement with the broader target audience. There is a need to assist countries to a greater extent in the development of national policies, their implementation and evaluation and to support them when undertaking future revisions. The focus group discussions confirmed this need.

This assessment has shown that the guidance has successfully reached a wider audience than the intended one. This observation may reflect the broadening range of stakeholders involved in health policy and decision making at least in some countries, as well as of healthcare workers involved in delivery of HIV testing services. The lessons learned from this evaluation should be used to inform future evaluations as well as the development of potential new guidance.

This evaluation had some limitations. Different sampling methods were used for the respondents of the two groups for the survey and for the focus groups, each with its own potential selection bias. Selection bias may have occurred when identifying participants, and despite a relatively high response rate, there is still an issue with non-respondents among the primary target audience, with those who are more familiar with the guidance potentially being more likely to respond to the survey. Acquiescence bias may also have occurred as the primary target audience was mainly comprised of ECDC contact points. The broader target audience was selected via convenience sampling through mailing list contacts and the number of responses was much lower than anticipated. The questions and response categories in the surveys were mostly pre-defined and some topics or issues may not have been captured or misinterpreted by the respondents. Finally, the results are primarily based on opinion and self-reported data in the survey responses. The citation review was limited as it was only performed in Scopus and Google Scholar. References to the guidance in national or sub-national policy documents or similar grey literature were therefore not captured so, the use of the guidance to inform national policy is likely to be under reflected in the citation review. The website access analysis was constrained by limited data availability due to a platform migration and other technical challenges, which again are likely to have resulted in an underestimation of the true number of page views and downloads.

### Conclusions

Three key elements emerged from this evaluation exercise: (i) the need to update the content of HIV testing guidance at more frequent intervals to capture evolving and accumulating evidence in the field, (ii) the value of engaging different constituencies and relevant organisations such as WHO, non-governmental and civil society organisations, clinical specialties and professional societies in a collaborative approach to foster HIV testing coverage and diversify the offer of testing modalities uptake and (iii) monitoring and evaluation of testing initiatives is considered an area for further development to devise a robust and standardised framework that can be used in the EU/EEA. Guidance of this sort should contribute to strengthen efforts to increase coverage, uptake of HIV testing and access to testing through diversification of delivery channels.
